# 3D photogrammetry quantifies growth and external erosion of individual coral colonies and skeletons

**DOI:** 10.1038/s41598-017-16408-z

**Published:** 2017-12-01

**Authors:** Renata Ferrari, Will F. Figueira, Morgan S. Pratchett, Tatiana Boube, Arne Adam, Tania Kobelkowsky-Vidrio, Steve S. Doo, Trisha Brooke Atwood, Maria Byrne

**Affiliations:** 1 School of Life and Environmental Sciences, Edgeworth David Building, Science Road, The University of Sydney, Sydney, NSW 2006 Australia; 2 Australian Institute of Marine Sciences, PMB No. 3, Townsville, Queensland 4810 Australia; 3 ARC Centre of Excellence for Coral Reef Studies, James Cook University, Townsville, Australia; 4 Department of Watershed Sciences and Ecology Center, Utah State University, Logan, UT USA; 5 Global Change Institute, The University of Queensland, St. Lucia, QLD Australia; 6 School of Medical Sciences, The University of Sydney, Sydney, NSW 2006 Australia

## Abstract

Growth and contraction of ecosystem engineers, such as trees, influence ecosystem structure and function. On coral reefs, methods to measure small changes in the structure of microhabitats, driven by growth of coral colonies and contraction of skeletons, are extremely limited. We used 3D reconstructions to quantify changes in the external structure of coral colonies of tabular *Acropora* spp., the dominant habitat-forming corals in shallow exposed reefs across the Pacific. The volume and surface area of live colonies increased by 21% and 22%, respectively, in 12 months, corresponding to a mean annual linear extension of 5.62 cm yr^−1^ (±1.81 SE). The volume and surface area of dead skeletons decreased by 52% and 47%, respectively, corresponding to a mean decline in linear extension of −29.56 cm yr^−1^ (±7.08 SE), which accounted for both erosion and fragmentation of dead colonies. This is the first study to use 3D photogrammetry to assess fine-scale structural changes of entire individual colonies *in situ*, quantifying coral growth and contraction. The high-resolution of the technique allows for detection of changes on reef structure faster than other non-intrusive approaches. These results improve our capacity to measure the drivers underpinning ecosystem biodiversity, status and trajectory.

## Introduction

Coral reefs are among the most vulnerable ecosystems to climate change, owing to the thermal sensitivity of scleractinian corals^1^, which bleach and can die when exposed to moderate increases in ocean temperatures. Increasing ocean temperatures, unequivocally linked to anthropogenic forcing of climate systems, are causing increasing frequency, severity and spatial extent of mass coral bleaching^2^. On Australia’s Great Barrier Reef, for example, climate induced coral bleaching emerged as the single biggest cause of coral loss in 2016^2^ and this is being compounded by further coral bleaching in 2017. Meanwhile, sustained increases in ocean temperatures are also contributing to declines in the growth and performance of individual coral colonies, thereby undermining the capacity of corals to withstand increasing incidence of major disturbances. In many locations, climate change is now the single greatest threat to the integrity and functioning of reef ecosystems^3^.

Scleractinian corals are the major autogenic habitat engineers on coral reefs, contributing to both habitat complexity^4,5^ and reef accretion^6^. Positive accretion of coral reef systems (net reef growth) is generally dependent upon high cover of living scleractinian corals^6^, and especially fast growing coral taxa, such as *Acropora* spp. In a warming ocean, strong storms and mass bleaching events are projected to occur more frequently^7–9^, with disproportionate impacts on *Acropora* corals^10^. It is also expected that there will be changes in topographic complexity of reef habitats^11^ as well as reduced rates of carbonate accretion^6,7,12^. Mass-bleaching and other biological disturbances (e.g. outbreaks of crown-of-thorns starfish) often cause widespread coral mortality, but unlike physical disturbances (e.g. severe tropical storms) do not have an immediate impact on the structural complexity of coral habitats^13^. Dead coral skeletons are however, subject to physical, chemical and biological erosion, and gradually contract or collapse over several years^4^.

Calcification and growth rates of scleractinian corals has attracted significant scientific interest since the late 1800’s, with a range of metrics being used to measure changes in size and weight of calcifying corals^14^. One of the most common metrics currently used for *in situ* studies is linear extension rate, which is often summarized as annual linear extension rate in centimeters per year for comparisons across studies^15^. Annual linear extension rate, however, does not fully account for the complex, three-dimensional (3D) growth of corals, nor does it measure change in volume or surface area, which have an important influence on the structure and function of reef habitats for important taxa such as reef fishes^16^. These limitations in accounting for fine-scale changes in carbonate accretion and erosion are partially overcome by explicitly quantifying changes in the weight and density of carbonate structures^17,18^, but current methods require manipulating corals and are overly invasive. For instance, buoyant weight method requires taking a coral colony out of the water, which may have negative impacts on its fitness. Given increasing threats to habitat-forming corals, there is an urgent need for a non-invasive method of comprehensively measuring changes in the size and structure of coral colonies *in situ*, which can be broadly applied across different coral morphologies over time^19^.

Recent advances in underwater photogrammetry enable 3D reconstructions from images of individual coral colonies to reef landscapes^20–22^. They can effectively be used to monitor corals over time without disturbing or manipulating them, and measuring external structural change of corals with higher precision than existing methodologies^22,23^. 3D reconstructions provide more robust metrics of coral growth and external skeletal erosion over time^11^. For instance, they allow the monitoring of the development of specific lesions or diseases on individual coral colonies through time, shedding light on the spatial dynamics of disease transmission among and within colonies^24^. Given that 3D technologies are now readily available to non-experts^20,25^, 3D reconstructions of corals can easily be achieved using off-the-shelf tools^20,25^.

3D reconstructions can provide highly accurate measures of the surface area and volume of coral colonies and skeletons across a broad range of morphologies^23^. Temporal comparisons can, in turn, provide reliable estimates of change in surface area and volume to document fine-scale patterns of structural change in individual coral colonies and skeletons. Implementation of these methods is urgently needed to quantify effects of sustained and ongoing disturbances on the structure and function of coral habitats^4,11,20,21,23^.

The aim of this study is to demonstrate and provide guidelines of how photogrammetry can be applied in the field to measure growth and contraction of corals (and other organisms). This study quantified fine-scale changes in the size and structure of both live and dead colonies of tabular *Acropora* (mostly, *Acropora hyacinthus*) over a 12 month period at Lizard Island, in the northern Great Barrier Reef, Australia. We used 3D reconstructions, with <2 mm accuracy (see methods and results), to test for changes in the linear extension, volume and 3D surface area of individual coral colonies and skeletons. We quantified both increases (growth) and decreases (hereafter referred to as external erosion) in the outer dimensions of individual coral colonies in 3D space. To enable comparisons with established metrics of coral growth we also calculated annual linear extension and carbonate erosion rates, using standardized measures of skeletal density for corals of the same genera and morphology, which were then compared to published estimates (http://coraltraits.org ^26^).

This study used tabular corals as a case study, but previous work^23^ has shown that 3D reconstructions can be applied to a broad range of coral morphologies with high-accuracy^23^. Our results contribute to marine ecology and environmental change science by: (1) operationalizing the application of a novel method to quantify key metrics on coral reef science and conservation: coral growth and skeletal contraction; (2) advancing our fundamental understanding of the growth and contraction dynamics of tabular corals and their skeletons, one of the most important ecosystem engineers in coral reefs^27–29^ and (3) providing a detailed workflow, and associated data, for any scientist to use 3D reconstructions to measure change in volume and surface area of individual marine or terrestrial organisms in a non-intrusive manner^11^. The key difference between this study and previous studies^20,22^ is that the method presented here does not require customized software and applies to organisms *in situ*.

## Results

### Precision assessment of 3D models

This study followed a very similar methodology to that in Figueira *et al*.^23^, which validated the accuracy and precision of metrics derived using this method for structure of corals of several morphologies (including tabular), and reported that the accuracy of 3D photogrammetry is <2 mm. Still, Figueira *et al*.^23^ used images taking in a swimming pool and different photogrammetric software (although the same algorithms) than we did. Thus, to confirm this precision applied to our models we randomly chose one live coral and one skeleton and built six 3D models for each in 2014.

The surface area had a coefficient of variation (CV) of <1.5% for both the live coral and the skeleton; while the volume had a CV of 2% for the live coral and 7% for the skeleton. The 7% was caused by one of the subset models (5), which presented quite a few tunnels through the top. If replicate 5 is excluded then the CV for volume is 3.1%. The tunnels were very obvious and would be easily detected by visually inspecting a model. Thus it is recommended that models are always visually inspected before performing any measurements, if errors are obvious the model should be discarded and rebuilt.

The deviation analysis in Geomagic is summarized by maximum, minimum and average errors. The average error amongst the 6 subset models in any one place was 1.3 ± 1.2 mm (mean ± SD) and 1.0 ± 1.2 mm, for the skeleton and live colony respectively. The maximum error amongst the 6 subset models was 34 mm for the skeleton and 32 mm for the coral colony; note that this value is driven by just one value across all the models. The average of the maximum error was 2.5 and 2% respectively, with an SD of about this same value. Given that the changes we have measured are an order of magnitude larger than these errors (change in volume and surface area of 20–50%), we can confirm that the precision of the 3D models utilized in this study is sufficient to measure annual coral growth and contraction.

### Change in coral colony volume and surface area

**Figure 1:**
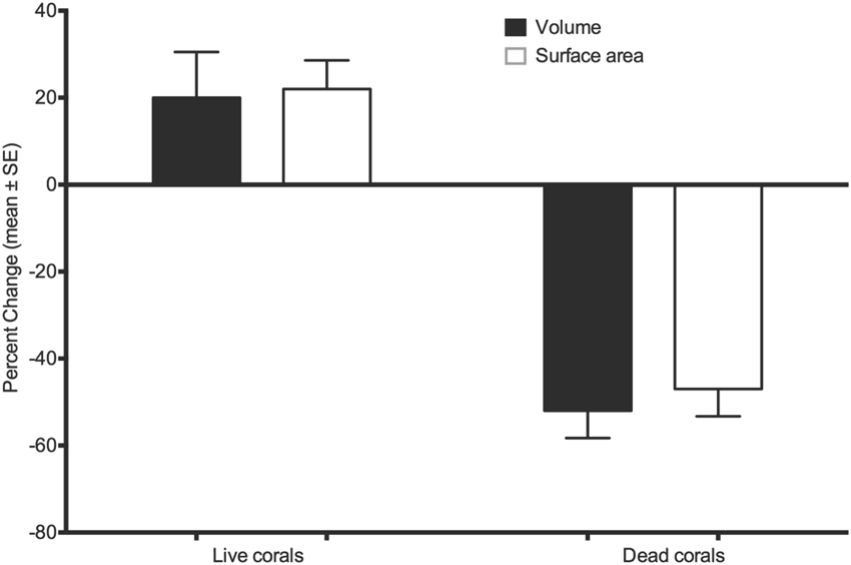
Standardized percent change in volume and surface area of 6 live and 7 dead tabular coral colonies between September 2014 and October 2015 in Lizard Island, Great Barrier Reef, Australia. The dashed line marks no change (0% change).

Of the 24 colonies of tabular *Acropora* spp. imaged in 2014, six living and seven dead colonies were available to re-sample (photographed) after 12 months, the others were dislodged by a cyclone (Tropical Cyclone Nathan, March 2015). The mean volume of live colonies (n = 6) increased by 20.5 ± 10.5% (mean ± SE), or 2,707 ± 1,509 cm^3^ during this period. Similarly, the mean surface area increased by 21.7 ± 6.6%, or 1,694 ± 729 cm^2^. On the other hand, the mean volume of dead colonies (n = 7) decreased by 52.2 ± 6.5%, equivalent to 4,973 ± 2,439 cm^3^ during the study period; and their mean surface area decreased by 47.1 ± 6.3%, or 2,507 ± 694 cm^2^ (Fig. 1).

### Change in annual linear extension

Annual linear extension rate was determined based on the maximum radius measured from the center of the colony to the farthest edge of the colony (see methods for details). The mean annual linear extension rate for live colonies was 5.62 ± 1.81 cm yr^−1^ (mean ± SE). Average annual linear extension for dead coral skeletons was −29.56 ± 7.08 cm yr^−1^ (mean ± SE) reflecting significant contraction in the outer dimensions.

### Estimated calcification rate

Based on the published skeletal density of *Acropora hyacinthus* in the Great Barrier Reef, 1.28 ± 0.034 g cm^−3^ (mean ± SE)^26^, and the increase in linear extension measured for live corals in this study, the estimated calcification rate was of 7.19 ± 2.32 g cm^−2^ yr^−1^ (mean ± SE) or 0.02 ± 0.006 g cm^−2^ day^−1^. It is important to consider that the published values of skeletal density are from a study with 6 samples of *A. hyacinthus* at 10 m depth. This is important because skeletal density may vary with environmental conditions, which would be different between our study and the referred study. Thus, the estimated calcification rate may not be accurate and we focus on mean annual linear extension for comparison.

### Identifying areas of change

**Figure 2:**
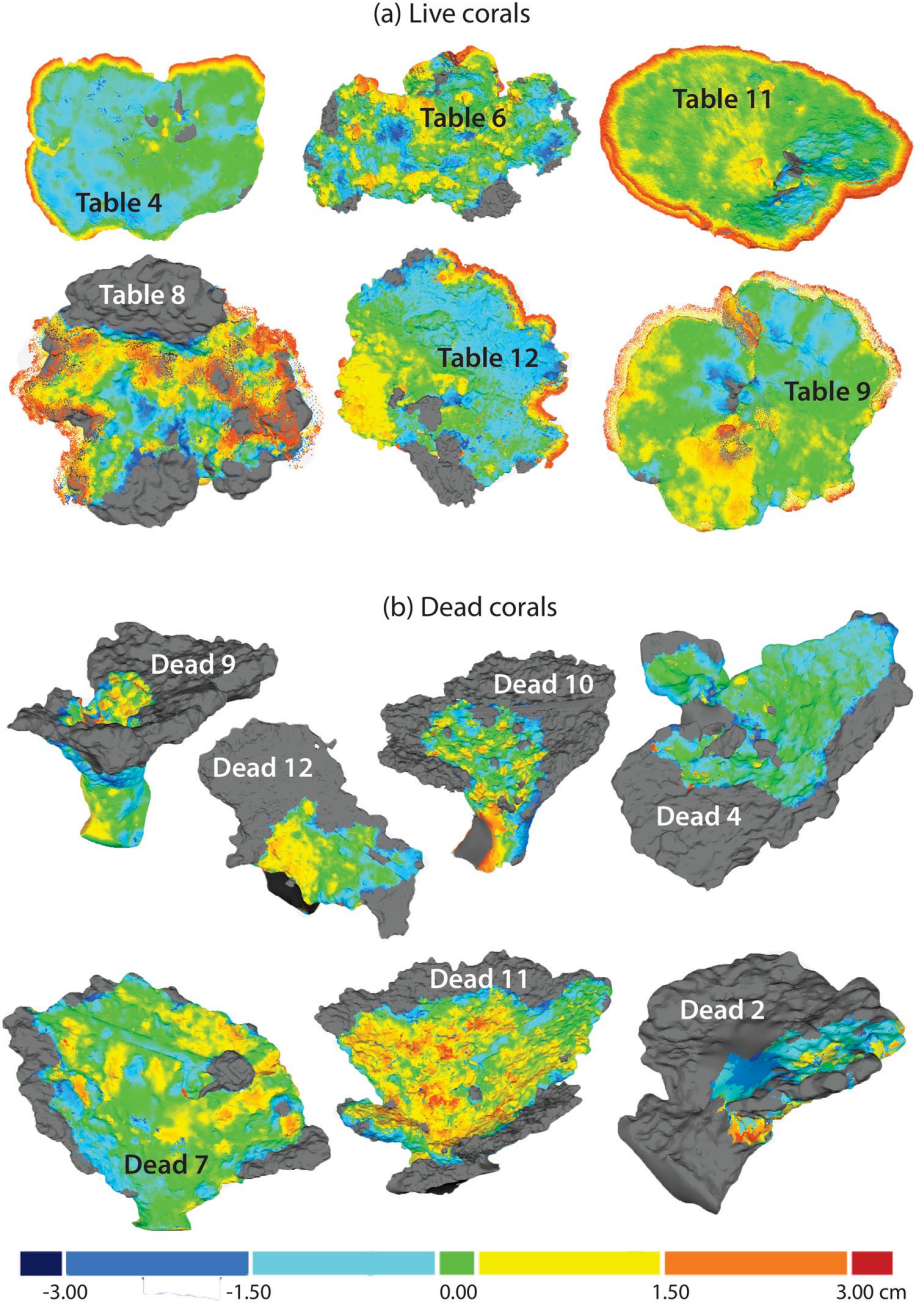
3D reconstruction comparison for six live (**a**) and seven dead (**b**) corals. Hot colors denote growth, while cool colors denote erosion (hot colors in dead corals represent algal overgrowth of skeletons). Gray areas are those that were only present on the 2014 reference reconstruction and not present in the 2015 reconstruction, and denote the difference between 2014 and 2015. Note the areas of dead tissue on live corals (**a**) probably due to fragmentation, also note the red near the center of coral colony “Table 9” (**a**), this is a whole that reduced in size due to coral growth during the study period. Note the light blue areas on live corals (**a**) are not due to composite results from multiple measures, they denote flattening of the plates as the corals grow due to fusion between branchlets. Note the warm colors on the skeletons (**b**) based on field observations these indicate algal growth on the skeletons in most cases.

Most of this growth occurred around the perimeter of the colonies (Fig. 2a). Growth was also observed around holes, for instance the coral colony labelled “Table 9” had a hole of 15 cm in diameter in 2014, which measured only 10 cm in diameter in 2015. The growth on the live coral stalks was small, 1.51 ± 0.96 cm yr^−1^ (mean ± SE). These stalks mostly increased in girth rather than length, showing that established colonies of tabular *Acropora* grow in 2 dimensions (Fig. 2a).

Skeletons contracted mostly around their perimeter, but not uniformly, with some areas of the perimeter eroding more than others (Fig. 2b). Branchlets over the surface of tabular *Acropora* corals also eroded externally within the 12-month observation period, contributing to declines in surface area and volume (Fig. 2b). Changes in the overall height of colonies was minimal, at an average of −0.25 cm yr^−1^ (±0.53 SE), (Fig. 1).

## Discussion

### Comparison of 3D metrics and conventional estimates

**Figure 3:**
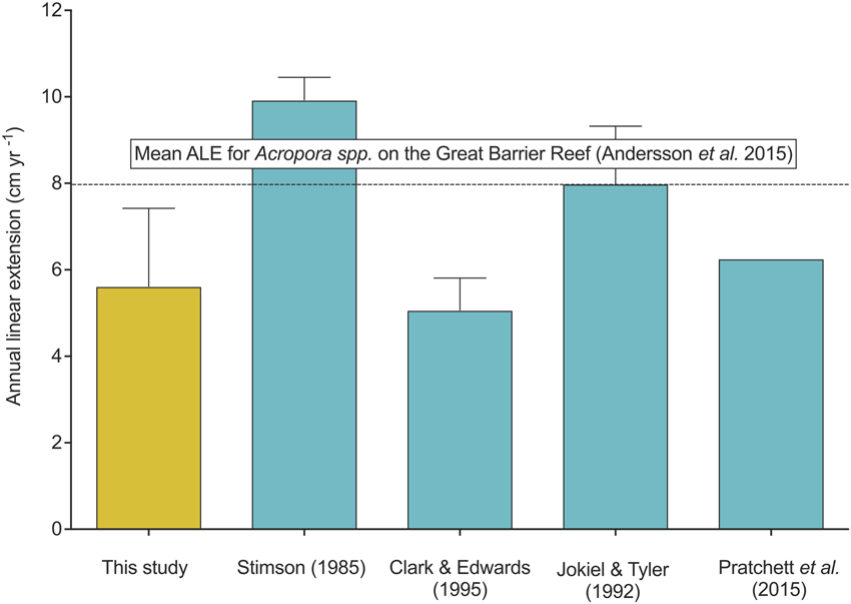
Comparison of (**a**) mean linear extension rate (ALE) and (**b**) mean annual erosion rates between this study and previous studies measuring growth of *Acropora hyacinthus* (source http://coraltraits.org ^26^). The dashed line (**a**) denotes the mean ALE for all *Acropora* spp. corals on the Great Barrier Reef^39^. Jokiel and Tyler^47^ refers to *A. hyacinthus, A. cytherea* and *A. divaricata*, the rest only *A. hyacinthus* annual linear extension. Data presented is restricted to tabular *Acropora* spp.

Our estimates of annual linear extension for live colonies (mean 5.62 ± 1.81 cm yr^−1^ ± SE) correspond closely with published estimates (Table 1, Fig. 3)^26^, revealing the utility of the 3D reconstruction approach to generate growth estimates (Table 1, Fig. 3). However, given the variation in published estimates of linear extension (Table 1), careful consideration in their interpretation is warranted. The time required to measure linear extension *in situ* is at beast comparable to the time required to image a coral colony underwater (a few minutes), and in some cases measuring linear extension *in situ* can take much longer. Yet, 3D metrics are moderately more costly and time consuming compared to conventional metrics such as annual linear extension, as they require an underwater camera, a computer, specialized 3D software and processing time.

**Table 1:** Summary of mean annual linear extension rates reported in previous studies.

	Species	Growth (cm yr^−1^)	Location	Reef type	Reference
Mean annual linear extension rate	*Acropora* spp.	5.62 (1.8 SE)	Lizard island, GBR	Lagoon	This study (2017)
	*A. hyacinthus*	9.41 (5.59 SD)	Marshall Islands	Lagoon	Stimson^27^
	*A. hyacinthus*	10.45 (8.79 SD)	Marshall Islands	Lagoon	Stimson^27^
	*A. cytherea*	6.67 (1.67 SD)	Johnston Atoll	NR	Jokiel & Tyler (1992)^47^
	*A. cytherea*	9.32 (3.18 SD)	Johnston Atoll	NR	Jokiel & Tyler (1992)^47^
	*A. hyacinthus*, *A. cytherea & A. divaricata*	4.15–5.81	Maldives	Reef flat	Clark and Edwards (1995)^48^
	*A. hyacinthus*	4.33 (0.29 SE)	Maldives	Reef flat	Clark & Edwards (1995)^48^
	*A. cytherea*	5.81 (8.5 SE)	Maldives	Reef flat	Clark & Edwards (1995)^48^
	*A. cytherea*	1.09 (0.199 SD)	Solitary Islands	NR	Harriott (1999)^49^
	*A. hyacinthus*	6.26 (NA)	NR	NR	Pratchett *et al*. (2015)^28^

Positive annual linear extension rates are only for *Acropora* spp. All studies were conducted at 0–6 m depth. NR: not reported, GBR: Great Barrier Reef. All extension rates were estimated using *in situ* measurements (i.e. calipers).

The adoption of a methodology is often dependent on its feasibility. Underwater cameras and computers are already part of most marine researcher’s tool box, and the specialized software can be obtained without cost (i.e. Visual SfM and Meshlab), for a modest price (i.e. Agisoft Photoscan) or for a high price (i.e. Geomagic). The main hurdle is the processing skill and time required to produce high quality 3D models, align models from different time steps and obtain relevant metrics. We solve much of these hurdles by providing guidelines on the recommended settings and computer specifications, which we have fine-tuned over 5 years of experience (Table 2). Ultimately, the feasibility of applying 3D photogrammetry to field studies will be determined by the number of models desired, the budget and the time frame of the project (Table 2). Metrics derived from 3D reconstructions are more precise and robust than field measurements such as linear extension. Moreover, they do not require handling of coral colonies underwater and allow documentation of novel metrics, such as volume, that might shed light on coral growth and external erosion/breakage patterns and dynamics^23^.

**Table 2:** Summary of processing settings used in Photoscan Professional (grey) (v1.1.6; Agisoft LLC 2015) and Geomagic Control (white) (2014 © 3D Systems) for image processing and comparisons of 3D reconstructions of coral colonies.

Step in workflow	Settings for each step	Time (min)
**Photoscan Pro processing**		
Image handling and quality control	Deleting blury images, loading images into PSP, creating chunks	5
Photo alignment	Accuracy high, pair preselection disabled, key point limit 40000, tie point limit 1000, do not constrain features by mask.	5
Dense Point Cloud	Quality medium, depth filtering mild, do not re-use depth maps.	5
Mesh	Surface type arbitrary, source data dense cloud, face count high, interpolation enabled, all point classes.	2
Texture	Mapping mode generic, texture from all cameras, blending mode mosaic, texture size 4096, no color correction.	3
Scaling	Create markers and scale bars manually in Photoscan Pro.	5
Export 3D reconstruction	Export as obj with texture.	1
**Geomagic Control processing**		
Cropping & cleaning	Manually using lasso tool for selecting and cropping, fill single hole for cleaning and filling holes.	5–10
Alignment	N-point alignment first, followed by the best-fit alignment.	1–2
Comparison	3D compare.- deviance analyses	1–2
Metric derivation	Volume and surface area are automatically derived with the analyses tools, radius and stalk length are manually derived with the measurement tool.	1–2
Total time per model		40–45

We were able to detect different patterns of growth, despite a low sample size, and showed how linear extension does not always accurately capture the growth, even for tabular corals, which have a simple morphology. For instance, colony 8 (Fig. 2a) was damaged by the cyclone, but only suffered partial mortality. Colony 8 lost ~25% of its surface area and volume on two damaged sides of its plate, but did grow on the two undamaged sides. It is probable that a biased measurement could have resulted if colony 8 had been traditionally measured using annual linear extension. Depending on exactly where on the colony plate linear extension was measured, this coral could have appeared as a coral that had increased or decreased in size during the study period. The 3D reconstruction for colony 8 revealed that while there was an increase in annual linear extension, the change in dimensions of this coral varied spatially across the colony. Thus, 3D reconstructions allow us to measure change spatially across the entire surface of a coral colony, and shed light on processes resulting in partial growth of some portions of a colony but no change or external erosion (physical injury or contraction) in other portions. Similarly, this technique can detect areas of external erosion (not just breakage) on both live and dead colonies, allowing for a spatially explicit assessment of structural change over time.

The use and utility of 3D photogrammetry for coral reef research has increased substantially in recent years^11,30–32^, though mostly for quantifying structural complexity of reef habitats at the scale of 10s–100s metres^22,33^. This is the first time that this technology has been used to quantify fine-scale structural changes of entire individual coral colonies *in situ*. Figueira *et al*.^23^ validated the accuracy and precision of metrics derived using this method for structure of corals of several morphologies (including tabular), and reported that the accuracy of 3D photogrammetry is <2 mm, which we confirmed in this study. Accordingly, we demonstrated how 3D reconstructions provide an unprecendented opportunity to accurately and comprehensively assess structural changes in the size and structure of individual corals, due to either growth and carbonate accretion by live corals or the loss of carbonate through chemical, biological and physical external erosion and breakage of dead skeletons.

Established methods for quantifying growth and carbonate accretion of scleractinian corals essentially focus on measuring either the overall mass of carbonate or skeletal material that is added through time (i.e., calcification^34^), or changes in overall colony dimensions (most often the ‘area of occupancy’^35^). While changes in the overall size of scleractinian colonies are fundamentally dependent on the deposition of calcium carbonate, the relationship between colony growth and calcification is complex^19^. This is especially the case for *Acropora* corals, where ongoing calcification may serve to increase skeletal density rather than overall colony dimensions^36^. Overall, calcification rates provide the most direct and readily comparable measure for assessing carbonate accretion, and are largely insensitive to differences in the morphology and predominant growth axis of different corals^19^. However, many ecological studies require explicit information on the size and structure (rather than mass) of coral colonies, which is generally inferred based on often crude and species-specific metrics^17,24,37^.

Here we show that 3D photogrammetry enables detailed insights into the changes in the colony dimensions of corals over time. We acknowledge the importance of expanding our approach to larger extents (both spatial and temporal), to include a larger sample size, other morphologies and genera of corals, and different reef types. We only applied 3D photogrammetry to one coral morphology, but past studies have shown that this technique can be applied to five other morphs^23^. Our results demonstrate the significant advantages that 3D photogrammetry has over many existing techniques used to measure coral growth and external erosion, such as branch extension^27^ or changes in planar area^38^. These advantages are mainly because 3D models provide a comprehensive picture of changes in colony size and shape in 3D while also providing a very effective method for quantifying changes in the volume and surface area of coral colonies and skeletons. Previously, such insights into patterns of growth for individual colonies were only possible by sacrificing whole colonies to visualise patterns of growth^39^. This technique (and other similar methods) also fail to account for any loss of skeletal material or external erosion, due to breakage or partial mortality^19^, which 3D photogrammetry quantifies.

It is well established that reef habitats may experience significant changes in large-scale topographic complexity due to the inevitable collapse of erect branching corals once dead^13,20,40,41^. However, this is the first study to measure external erosion of tabular *Acropora* spp. corals *in situ* and at time frames (months to years) that are consistent with most monitoring and research projects. Previous estimates of erosion of dead coral skeletons are based on the deployment of standardized blocks of carbonate cut from *Porites* spp. colonies. Given previous studies used standardized blocks it is not possible to directly compare our external erosion rates using the surface area-to-volume ratio relationship, which would predict highly complex skeletons would be more vulnerable to external erosion and decomposition compared to massive colonies. Furthermore, the skeletal density of *Porites* spp. is higher than that of *Acropora* spp. skeletons, and as such it is not possible to compare previously reported erosion rates to the external erosion rates measured in this study. We are confident on our metrics given that the annual linear extension rates for live corals were similar to those previously reported and the technique applied to both dead and live colonies is exactly the same.

This study provides a baseline of external erosion rate for tabular skeletons on leeward reefs, but further studies comparing the external erosion rates of undisturbed skeletons *in situ* are required to fully understand the probabilities and rates of reef flattening by reefs dominated by different morphologies (i.e. massive vs. tabular). Interestingly, the external erosion rates of the dead skeletons (−50%) in this study are more than double the growth rates of the live corals (20%). This suggests that while tabular corals grow relatively fast (especially compared to other corals^19^); they may also be susceptible to very rapid rates of external erosion and physical breakage (i.e. due to storm damage). Previous findings have highlighted the ongoing global decline in growth and calcification rates of modern reefs, as well as an increase in the frequency and strenght of tropical storms^17,42,43^. Approaches such as the one presented here have enourmous potential to help understand how different coral morphologies contribute to reef structural changes and thereby influence habitat structural complexity.

Given the significant threat posed by environmental change to both the persistence and performance of scleractinian corals^6,44,45^ as well as reef growth and structure^6,46^, there is a need for long-term studies to characterize changes in the growth and external erosion of individual coral colonies and skeletons. Development of 3D photogrammetry applications is an effective and timely innovation that allows for non-intrusive and repeated measurements of colony size and shape, and simultaneoulsy quantifies both coral construction (growth and carbonate accretion) and contraction (injuries to live corals, external erosion and brekage of exposed skeletons) of skeletal material. It is crucial to precisely quantify coral colony growth and external erosion rates to improve our ability to predict how ecological processes and biotic assemblages respond to habitat loss, especially in the face of acute and cumulative disturbances^10,11^. In turn, metrics such as the ones produced here could inform important mechanistic models capable of predicting coral reef resilience or collapse in response to climate change.

## Methods

### Coral sampling

Twelve dead and twelve live tabular corals were randomly selected within back reef habitat at Lizard Island (−14.685897°, 145.442058°), on the Great Barrier Reef, Australia. All corals were on sheltered patch reefs between 0–6 m depth. For each coral, a ruler was placed nearby for scale and a hemispherical pattern was used to photograph it. Depending on the coral size, which ranged between 1000–20000 cm^2^ in surface area (10–180 cm in diameter), a total of 52–109 images were taken (larger corals required more images). All colonies were imaged during daylight hours (for details of how to image a coral colony see^23^), and Geographic Positioning System coordinates were taken.

### Image acquisition

Images were captured by a snorkeler using a digital camera (24 MP Sony^®^ NEX-7) in a Nauticam housing with a fixed Sony 16 mm f/2.8 E-mount wide-angle lens, with natural lighting. Prior to photographing individual colonies, a relevant scale (ruler or Rubik’s Cube^®^) was placed immediately adjacent to the colony. Each colony and relevant scale reference was then photographed capturing overlapping photographs at a rate of ~1 photo every 1–2 seconds from different viewpoints using a standardized pattern. The pattern consisted of eight consecutive arched passes at approximately 50–100 cm from the coral colony, each pass captured a series of overlapping images from top to bottom covering the entire surface of the coral and captured 5–10 photographs with ~80% overlap. The camera was moved ~45° to start the next pass, following the eight arched passes two final 360° spiral passes around the colony (~10–20 images per pass) captured more images. Image acquisition took ~5 minutes per coral. Coral colonies were initially imaged in September 2014 and re-imaged in October 2015. However, cyclone Nathan (March 2015) killed six of the live colonies and dislodged five of the dead colonies. As a result, of the original 24 colonies only six live and seven dead colonies were re-imaged in 2015.

### Image processing to produce 3D reconstructions

Twenty-six 3D reconstructions (13 corals at each of two time steps) were built using Photoscan Professional (v1.1.6; Agisoft LLC 2015, S1 Virtual 3D reconstructions here, Table 2). First, images were revised and bad quality images were removed (blurry, dark or with moving objects between the camera and the colony), as they added noise during the model reconstruction process. Photoscan Pro photo alignment generates tie points between photos, which aligns them over the object to be modelled and estimates the camera position for all photos. Building of the dense point cloud triangulates from the tie points to identify and locate in space unique pixels that are found in overlapping photos. Meshing interpolates surface areas around the points generated in the dense point cloud. Finally, texturing overlays the color and texture from the pixels in the original photos on to the mesh according to the original camera alignment (Table 2).

The average time used to reconstruct one digital models was ~15 min on a Windows 10 workstation (specifications of 64 bit OS, Dell Optiplex 7040, Intel core i7-6700 @ 3.4 GHz, 32 GB RAM, 1 TB HDD, Graphics: AMD Radeon 32 GB). On a network of 5 computers (similar specifications) the processing of one coral colony took ~5 min (see network processing in PhotoscanPro user manual). This technique has a 3D distance precision of 1.7 ± 1.2 mm (mean ± SD) for tabular corals^23^. Once all 26 3D reconstructions were processed, they were scaled in Photoscan Pro by using the ruler and/or the Rubik’s Cube^®^ to create at least two scalebars per model. After scaling each reconstruction was exported as an.obj file for importing, cleaning and comparing.

### Precision assessment of 3D models

To confirm the precision reported by Figueira *et al*.^23^ applied to our models, we randomly chose one live coral and one skeleton and built six 3D models for each in 2014. To do so we randomly removed 10% of photos from each data set, built the model as per Table 2 settings in PhotoscanPro, measured surface area and volume, and simultaneously compared the six models against each other in Geomagic using the *average model* tool, which resulted in a deviation analyses. The deviation analysis in Geomagic, overlays all models in 3D and measures the absolute difference in distance between the points on any one model and the points on the model that is closest to the average model. The difference in absolute distance is measured for each of thousands of points on each model, and then summarized by maximum, minimum and average error for the deviation analyses. This was done separately for the live coral colony and the skeleton.

### 3D reconstructions comparison over time

We used Geomagic Control (2014 © 3D Systems) to compare the 3D reconstructions from 2014 to 2015. We aligned the two models, deleted all unnecessary data (*e.g*. substrate around each colony), and manually closed each 3D reconstruction by filling any small holes on the 3D reconstruction, which were present if occlusions occurred during the imaging process. For instance, it is not possible to photograph the base of the stalk of a tabular coral, given that it is attached to the reef substrate, so this hole was filled using a flat plane. Within Geomagic Control, model alignment was performed using the *N-point alignment* first, followed by the *best-fit alignment* functions. Once aligned, the coral colony was selected using the *lasso* tool and unnecessary data was deleted. Then, the 3D reconstruction was closed manually using the *fill single* function. Reconstructions of the same colony across time steps were compared using the *3D compare* function, and metrics for volume and surface area were quantified using the *analyses* tools.

### Derived metrics of volume, 3D surface area, linear extension, calcification rate and data analyses

3D surface area and volume were calculated for each 3D reconstruction and time point. To compare our metrics with previous studies, and to better understand how growth and erosion influence the morphology of tabular corals, we obtained two additional metrics from each 3D reconstruction pair: annual linear extension rate of the colony (cm yr^−1^) and length of the stalk (cm yr^−1^). These two additional metrics were derived manually from the overlaid 3D reconstruction pairs using the *measurement* tool in Geomagic Control. Both linear extension and stalk length were measured on each 3D model when the two 3D models for 2014 and 2015 were overlapped, this would be equivalent to having a permanent marker in the field from where to measure linear extension at each time step. The change in volume and 3D surface area was standardized by size and calculated as a proportional change (percentage). The decrease in volume and surface area is referred to as external erosion. We used published skeletal density values^26^ and our metric of annual linear extension to calculate the average annual calcification rate of the live corals in our study as per equation (1).

1 $${Annual}\,{Calcification}\,{Rate}={Skeletal}\,{Density}\ast {{ALE}}_{{\rm{change}}}$$

where *Skeletal Density* is the published value (1.28 g cm^−3^)^26^, and *ALE*_*change*_ is the change in mean annual linear extension measured in this study (5.62 cm).

### Data availability

The models generated by this study are available in: https://sketchfab.com/renataferrari/collections/3d-models-to-measure-coral-growth-and-erosion.
